# Geographical and Tick-Dependent Distribution of Flavi-Like Alongshan and Yanggou Tick Viruses in Russia

**DOI:** 10.3390/v13030458

**Published:** 2021-03-11

**Authors:** Ivan S. Kholodilov, Oxana A. Belova, Evgeny S. Morozkin, Alexander G. Litov, Anna Y. Ivannikova, Marat T. Makenov, Alexey M. Shchetinin, Sergey V. Aibulatov, Galina K. Bazarova, Lesley Bell-Sakyi, Liubov A. Bespyatova, Sergey V. Bugmyrin, Nikita Chernetsov, Liubov L. Chernokhaeva, Larissa V. Gmyl, Anna N. Khaisarova, Alexei V. Khalin, Alexander S. Klimentov, Irina V. Kovalchuk, Svetlana V. Luchinina, Sergey G. Medvedev, Alexander A. Nafeev, Natalia D. Oorzhak, Elena V. Panjukova, Alexandra E. Polienko, Kristina A. Purmak, Evgeniya N. Romanenko, Evgeniy N. Rozhdestvenskiy, Anna A. Saryglar, Anton F. Shamsutdinov, Nataliya I. Solomashchenko, Vladimir A. Trifonov, Evgenii G. Volchev, Pavel G. Vovkotech, Alexander S. Yakovlev, Olga B. Zhurenkova, Vladimir A. Gushchin, Lyudmila S. Karan, Galina G. Karganova

**Affiliations:** 1 Laboratory of Biology of Arboviruses, “Chumakov Institute of Poliomyelitis and Viral Encephalitides” FSBSI “Chumakov FSC R&D IBP RAS”, 108819 Moscow, Russia; ivan-kholodilov@bk.ru (I.S.K.); mikasusha@bk.ru (O.A.B.); novosti-wxo@yandex.ru (A.G.L.); strannotut@gmail.com (A.Y.I.); dec151ll@mail.ru (L.L.C.); lvgmyl@mail.ru (L.V.G.); polienko.ae@yandex.ru (A.E.P.); alex-montreal@yandex.ru (A.S.Y.); 2 Department of Molecular Diagnostics and Epidemiology, Central Research Institute of Epidemiology, 111123 Moscow, Russia; evgeny.morozkin@mail.ru (E.S.M.); makenov@cmd.su (M.T.M.); zhurenkova@cmd.su (O.B.Z.); karan@cmd.su (L.S.K.); 3 Pathogenic Microorganisms Variability Laboratory, Gamaleya Federal Research Centre for Epidemiology and Microbiology, Ministry of Health of the Russian Federation, 123098 Moscow, Russia; shchetinin.alexey@yandex.ru (A.M.S.); wowaniada@gmail.com (V.A.G.); 4 Laboratory of Parasitic Arthropods, Zoological Institute, Russian Academy of Sciences, 199034 St. Petersburg, Russia; s.v.aibulatov@gmail.com (S.V.A.); hallisimo@yandex.ru (A.V.K.); smedvedev@zin.ru (S.G.M.); 5 Laboratory of Bacteriology, Altai Antiplague Station of Rospotrebnadzor, 649000 Gorno-Altaisk, Russia; altai-chuma@mail.ru; 6 Department of Infection Biology and Microbiomes, Institute of Infection, Veterinary and Ecological Sciences, University of Liverpool, Liverpool L3 5RF, UK; l.bell-sakyi@liverpool.ac.uk; 7 Laboratory for Animal and Plant Parasitology, Institute of Biology of Karelian Research Centre, Russian Academy of Sciences (IB KarRC RAS), 185910 Petrozavodsk, Russia; gamasina@mail.ru (L.A.B.); sbugmyr@mail.ru (S.V.B.); 8 Laboratory of Ornithology, Zoological Institute, Russian Academy of Sciences, 199034 St. Petersburg, Russia; nikita.chernetsov@gmail.com; 9 Department of Vertebrate Zoology, St. Petersburg State University, 199034 St. Petersburg, Russia; 10 Center for Hygiene and Epidemiology in the Ulyanovsk Region, 432005 Ulyanovsk, Russia; an_stolyarova@mail.ru (A.N.K.); nafeev@mail.ru (A.A.N.); vovkotech87@mail.ru (P.G.V.); 11 Laboratory of Biochemistry, “Chumakov Institute of Poliomyelitis and Viral Encephalitides” FSBSI “Chumakov FSC R&D IBP RAS”, 108819 Moscow, Russia; aklimentov@mail.ru; 12 Laboratory of Biology and Indication of Arboviruses, Department Ivanovsky Institute of Virology, Gamaleya Federal Research Centre for Epidemiology and Microbiology, Ministry of Health of the Russian Federation, 123098 Moscow, Russia; 13 Office of Rospotrebnadzor in the Stavropol Territory, 355008 Stavropol, Russia; kovalchuk_IV@26.rospotrebnadzor.ru (I.V.K.); sni@fbuz26.ru (N.I.S.); 14 Stavropol State Medical University, 355017 Stavropol, Russia; 15 Office of Rospotrebnadzor in the Chelyabinsk Region, 454092 Chelyabinsk, Russia; luchinina_sv@74.rospotrebnadzor.ru; 16 Infectious Disease Hospital, 667003 Kyzyl, Russia; natalia.oorzhak@yandex.ru (N.D.O.); anna_kyzyl@mail.ru (A.A.S.); 17 Institute of Biology, Komi Science Center, Ural Branch of Russian Academy of Sciences, 167982 Syktyvkar, Russia; panjukova@ib.komisc.ru; 18 FBIH “Center for Hygiene and Epidemiology in the Stavropol kray”, 355008 Stavropol, Russia; kristypurmak.ru@mail.ru (K.A.P.); poliost@fbuz26.ru (E.N.R.); 19 Director, Altai Antiplague Station of Rospotrebnadzor, 649000 Gorno-Altaisk, Russia; chumagorny@mail.ru; 20 Kazan Scientific Research Institute of Epidemiology and Microbiology of Rospotrebnadzor, 420015 Kazan, Russia; shamsutdinov2006@yandex.com (A.F.S.); vtr-08@mail.ru (V.A.T.); 21 Kazan State Medical Academy—Branch Campus of the FSBEI FPE «Russian Medical Academy of Continuous Postgraduate Education» of the Ministry of Healthcare of the Russian Federation, 420012 Kazan, Russia; 22 Institute of Living Systems Immanuel Kant Baltic Federal University, 236041 Kaliningrad, Russia; e.volchev@hotmail.com; 23 Faculty of Biology, Lomonosov MSU, 119991 Moscow, Russia; 24 Institute for Translational Medicine and Biotechnology, Sechenov University, 119146 Moscow, Russia

**Keywords:** Alongshan virus, Yanggou tick virus, Jingmen tick virus, *Flavivirus*, flavi-like virus, segmented virus, amino acid substitutions, tick cell line

## Abstract

The genus *Flavivirus* includes related, unclassified segmented flavi-like viruses, two segments of which have homology with flavivirus RNA-dependent RNA polymerase NS5 and RNA helicase-protease NS3. This group includes such viruses as Jingmen tick virus, Alongshan virus, Yanggou tick virus and others. We detected the Yanggou tick virus in *Dermacentor nuttalli* and *Dermacentor marginatus* ticks in two neighbouring regions of Russia. The virus prevalence ranged from 0.5% to 8.0%. We detected RNA of the Alongshan virus in 44 individuals or pools of various tick species in eight regions of Russia. The virus prevalence ranged from 0.6% to 7.8%. We demonstrated the successful replication of the Yanggou tick virus and Alongshan virus in IRE/CTVM19 and HAE/CTVM8 tick cell lines without a cytopathic effect. According to the phylogenetic analysis, we divided the Alongshan virus into two groups: an *Ixodes persulcatus* group and an *Ixodes ricinus* group. In addition, the *I. persulcatus* group can be divided into European and Asian subgroups. We found amino acid signatures specific to the *I. ricinus* and *I. persulcatus* groups and also distinguished between the European and Asian subgroups of the *I. persulcatus* group.

## 1. Introduction

The genus *Flavivirus* belongs to the family *Flaviviridae*, whose representatives are small enveloped viruses with ssRNA(+) genomes of about 10 kb in length that encode one large open reading frame [1]. In the last decade, flavi-like viruses with a genome length exceeding 16 kb [2] and segmented genome [3] have been found. Segmented flavi-like viruses were included in the group of unclassified viruses, related to the genus *Flavivirus* [4].

The representatives of the Jingmenvirus (JMV) group, such as the Jingmen tick virus (JMTV), Alongshan virus (ALSV), Yanggou tick virus (YGTV) and others, have segmented ssRNA(+) genomes, two segments of which have homology with the well-studied flavivirus RNA-dependent RNA polymerase NS5 (segment 1) and RNA helicase-protease NS3 (segment 3) [3,5]. The remaining two segments are specific only to JMVs and encode glycoproteins—a novel upstream open reading frame (nuORF), VP1a and VP1b proteins (segment 2) [5,6,7] and other virus proteins—VP2 and VP3 (segment 4) [2,3,5]. Excluding plant viruses, before the discovery of JMTVs [3], only representatives of the genus *Omegatetravirus* of the family *Tetraviridae*, which infect insects [8], and the genera *Alphanodavirus* and *Betanodavirus* of the family *Nodaviridae*, which infect insects and fish, respectively [9], were known to have a segmented ssRNA(+) genome.

Representatives of the JMV group are found on almost all continents: Eurasia [2,3,5,7,10,11,12,13], Africa [11,14], South America [11,14,15,16,17] and North America [18]. RNA of this virus group has been detected in different species of insects (fruit flies [19] and mosquitoes [20]), ticks [2,3,5,7,10,11,13,17,20] and mammals [3,11,14,15,21], including humans [10,12,20].

The first detection of ALSV occurred in China in a patient with fever of unknown aetiology. Later, this virus was detected in mosquitoes [20], ticks [7,11,13,20] and mammals [20,22]. We showed, for the first time, that the virus can reproduce and cause persistent infection in tick cell cultures [7].

YGTV was first detected in *Dermacentor nuttalli* ticks in China and was shown to be closely related to ALSV [13]. There is currently no further published information about the distribution of this virus and its properties.

The JMV group is currently receiving increased attention, because, on the one hand, the study of this group provides new information about the variety of possibilities for genome organisation in RNA(+) viruses and their evolution. On the other hand, the uncertainty in host range and virulence in humans and animals requires investigation of the epidemiological potential of the JMV group. An important stage in the study of these viruses is the assessment of their distribution and delineation of the host range.

In the present study, we describe the distribution of ALSV and YGTV in Russia and their phylogenetic relationships.

## 2. Materials and Methods

### 2.1. Collection and Processing of Mosquitoes

**Figure 1:**
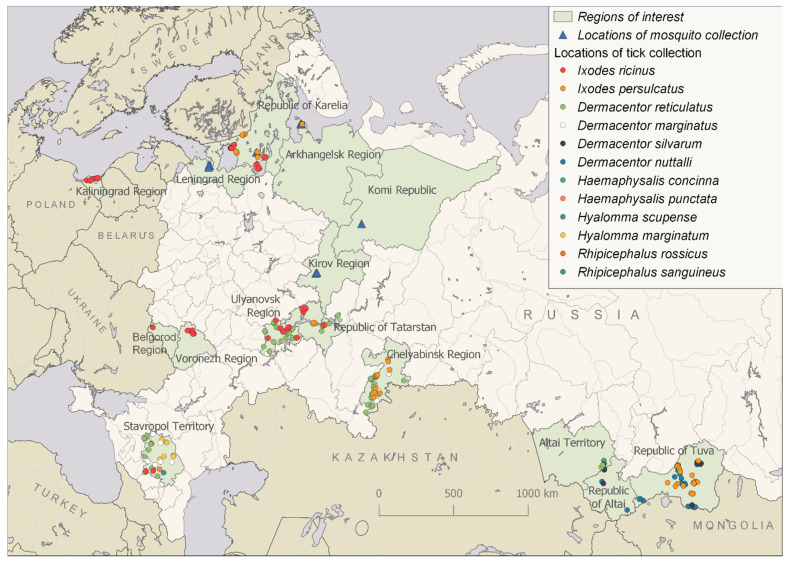
Locations of tick and mosquito collections in different regions of Russia.

Two methods were used to collect mosquitoes: collection of attacking adult females with an aspirator (Kirov Region and Komi Republic) [23] or with a Krishtal trap [24] (Arkhangelsk Region and the Republic of Karelia) and the collection of larvae and pupae from temporary and permanent reservoirs (Leningrad Region and St. Petersburg). Collection and rearing of larvae and pupae of mosquitoes was carried out according to the method described previously [25]. After hatching, adults were placed at −20 °C for 1–5 min. Immediately after this, genera and species determination was carried out using identification keys [23,26,27]. The locations of mosquito collection, species and their abundance are presented (Figure 1 and Table S1).

Adult mosquitoes were homogenised in pools of 15–17 individuals according to species composition, location, site and method of collection using the laboratory homogeniser TissueLyser II (QIAGEN, Hilden, Germany) in Medium 199 with Earle’s salts (FSBSI Chumakov FSC R&D IBP RAS, Moscow, Russia).

### 2.2. Collection and Processing of Ticks

Ticks were collected by flagging from vegetation and manually from domestic animals, including cows, sheep, horses and dogs. Ticks were identified using taxonomic keys [28,29]. The locations of tick collections and tick species are presented (Figure 1 and Table S2).

Adult ticks were homogenised individually or in pools (three to five individuals) according to species composition, location, site and method of collection using the laboratory homogeniser in Medium 199 with Earle’s salts. To analyse ticks individually, we added 300 μL of the medium per *Ixodes* spp. tick and 500 μL per *Dermacentor*, *Hyalomma*, *Haemaphysalis* or *Rhipicephalus* spp. tick. When ticks were analysed in pools, the volume of the medium added was calculated according to the species and the number of ticks in the pool, as follows: for each *Ixodes* spp. tick, 150 μL of medium was added; for each tick of the other species, 200 μL of medium was added.

### 2.3. Infection of Tick Cell Lines

We used cell lines derived from embryos of the ticks *Ixodes ricinus* (IRE/CTVM19) [30] and *Hyalomma anatolicum* (HAE/CTVM8) [31] provided by the Tick Cell Biobank (Liverpool, UK). Both tick cell lines were maintained at 28 °C in L-15 (Leibovitz) medium (FSBSI Chumakov FSC R&D IBP RAS, Moscow, Russia) supplemented with 10% tryptose phosphate broth (Difco, Detroit, MI, USA), 20% foetal bovine serum (Gibco, Invitrogen, Carlsbad, CA, USA), 2-mM l-glutamine and antibiotics, as described earlier [32]. Prior to infection, IRE/CTVM19 or HAE/CTVM8 cells were seeded in flat-sided culture tubes (Nunc, ThermoFisher Scientific, Waltham, MA, USA) in 2.2 mL of complete medium and incubated at 28 °C. A week later, cells were infected by adding 200 μL of virus-containing material (unfiltered tick homogenate or culture supernate) and incubated at 28 °C. Medium was changed at weekly intervals by removal and replacement of 1.1 mL; the spent medium was used to harvest the virus, as described below. Subcultures were carried out occasionally, as described previously [7].

For high-throughput sequencing, the infected IRE/CTVM19 culture supernate (spent medium) was clarified by centrifugation at 10,000 rpm for 30 min at 4 °C using an SW-28 rotor in an Optima L-90K Ultracentrifuge (Beckman Coulter, Brea, CA, USA) and was then ultracentrifuged at 25,000 rpm for 6 h at 4 °C using the same rotor. The resultant pellet was processed as described below in Section 2.5.

### 2.4. Reverse-Transcriptase PCR (RT-PCR) and Sequencing of Amplified Products

Viral RNA from tick suspensions, mosquito suspensions and infected cell culture supernate was isolated with TRI Reagent LS (Sigma-Aldrich, MO, USA) according to the manufacturer’s protocols. Reverse transcription was performed with random hexamer primers (R6) and M-MLV reverse transcriptase (Promega, Madison, WI, USA), according to the manufacturer’s protocols. Viral genomic cDNA was amplified by PCR using primers for the genus *Flavivirus* [33] and specific primers for ALSV: Alongshan_seg1_5 and Miass_NS5_1R for segment 1 (Table S3) and Miass_gly_3F and Miass_gly_3R for segment 2 [7]. Sequencing was carried out in both directions directly from PCR-amplified DNA on the ABI PRISM 3730 (Applied Biosystems, Foster City, CA, USA) sequencer using ABI PRISM^®^ BigDye™ Terminator v. 3.1. Genomic sequences were assembled using SeqMan software (DNAstar, Madison, WI, USA).

### 2.5. High-Throughput Sequencing

Total RNA was extracted from the ultracentrifugation pellet using TRI Reagent LS. RNA was fragmented and reverse-transcribed into cDNA with R6 primers using RevertAid reverse transcriptase (ThermoFisher Scientific, Waltham, MA, USA), followed by second-strand synthesis with the NEBNext Ultra II Non-Directional RNA Second-Strand Synthesis Module (New England Biolabs Inc., Ipswich, MA, USA). Resultant double-stranded DNA was purified using Ampure XP (Beckman Coulter, Brea, CA, USA) and subjected as an input for library preparation using the NEBNext^®^ Fast DNA Library Prep Set for Ion Torrent™ (New England Biolabs Inc., Ipswich, MA, USA) following the manufacturer’s instructions. The resultant DNA library was quantified with the Ion Library TaqMan™ Quantitation Kit (ThermoFisher Scientific, Waltham, MA, USA), followed by templating on the Ion Chef instrument (ThermoFisher Scientific, Waltham, MA, USA) and sequencing on the Ion S5XL instrument with the viral library constituting a part of the Ion 530 Chip. Raw reads were filtered by quality (q20) and length (>35) using Trimmomatic v.0.39 [34] and mapped on the strain Miass527 genome using the UGENE v1.32.0 “map NGS Reads to Reference” function with 10% of mismatches allowed. After mapping, virus consensus sequence was exported using default UGENE settings [35].

### 2.6. Phylogenetic Analysis

RNA sequences of all published full genomes of strains of ALSV, YGTV and representatives of JMTV (27 January 2021), and all the amplicons (from samples in which the virus was detected but not cultured) and cultured strains described in this article were used in the phylogenetic analysis. The nucleotide sequences of the genome coding regions of segment 1 and segment 2 were aligned separately using ClustalW. Phylogenetic analysis of the fragments of segments 1 and 2 was conducted using the neighbour-joining method in MEGA X with 1000 bootstrap replications.

To identify the open reading frames in segment 2 of ALSV, we used the Snap Gene Viewer program with translation options: minimum length 75 amino acids, selected options “Require a start codon ATG” and “except at DNA ends” and “Standard” options of Genetic code for ORFs and new features. Complete amino acid sequences of VP1a, VP1b and the novel upstream open reading frame (nuORF) [7] were used for phylogenetic analysis using the neighbour-joining method in MEGA X with 1000 bootstrap replications.

## 3. Results

### 3.1. Collection and Screening for Flavi-Like Viruses of Mosquitoes

We collected 4409 mosquitoes belonging to 12 species: Aedes cinereus, Aedes cantans, Aedes communis, Aedes punctor, Aedes diantaeus, Aedes pionops, Culex pipiens, Culex territans, Anopheles messeae, Anopheles claviger, Culiseta morsitans and Culiseta bergrothi (Figure 1 and Table S1). Some mosquitoes were captured as adults (Kirov Region, Komi Republic, the Republic of Karelia and Arkhangelsk Region), whereas others were caught at the larval stage, with the subsequent transformation into adults achieved in the laboratory (Leningrad Region and St. Petersburg). We did not detect any flavi-like viruses in mosquitoes using the panflavi primers described earlier [33].

### 3.2. Alongshan Virus Detection in Ticks, Isolation and Phylogenetic Relationships

**Figure 2:**
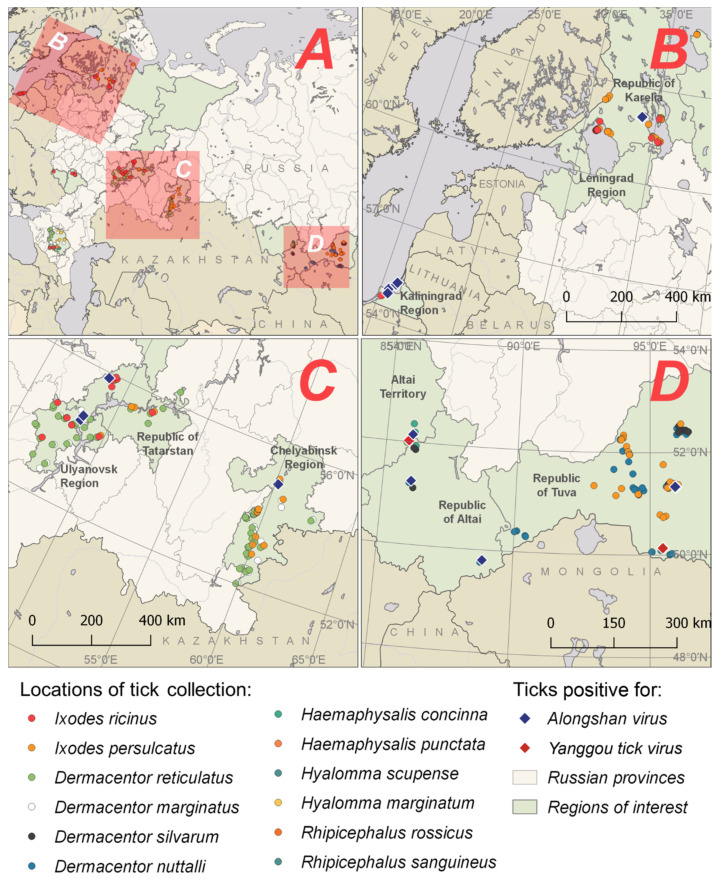
Locations of the Alongshan virus and Yanggou tick virus detection in ticks collected from vegetation and animal hosts in different regions of Russia. (**A**) Location of sample areas shown in detail in maps B, C and D; (**B**–**D**) Locations where ticks were collected.

We collected ticks in different regions of Russia during the period 2011–2019 (Figure 1 and Figure 2 and Table S2). A total of 7122 ticks of 12 species were collected and analysed: *I. ricinus*, *Ixodes persulcatus*, *Dermacentor reticulatus*, *Dermacentor marginatus*, *Dermacentor silvarum*, *D. nuttalli*, *Hyalomma marginatum*, *Hyalomma scupense*, *Haemaphysalis concinna*, *Haemaphysalis punctata*, *Rhipicephalus rossicus* and *Rhipicephalus sanguineus*.

We detected RNA of ALSV in 44 individuals or pools of various tick species collected from vegetation (Figure 2 and Table 1). Twelve positive samples were detected with panflavi primers [33], twenty eight positive samples with specific primers Miass_gly_3F and Miass_gly_3R (Table S3) and four positive samples with Alongshan-OUT-F and Alongshan-OUT-R (Table S3). The virus was found in almost all studied regions, but its distribution was very uneven. Many positive samples were found at single sites of tick collection in the Kaliningrad (three samples) and Chelyabinsk (nine samples) Regions and a few at sites in the Ulyanovsk Region (one sample) and the Republics of Altai (two samples), Tatarstan (one sample), Karelia (two samples) and Tuva (one sample). We did not detect ALSV in the Belgorod, Voronezh or Stavropol Regions. ALSV was detected in *I. persulcatus* (Republics of Tuva, Karelia and Chelyabinsk Region); *I. ricinus* (Kaliningrad and Ulyanovsk Regions and the Republic of Tatarstan); *D. reticulatus* (Ulyanovsk Region); *D. nuttalli* (Republic of Altai) and *H. concinna* (Altai Territory) (Table 1).

The number of analysed ticks was insufficient to calculate the virus prevalence in some regions. Therefore, the infection rate of ticks was estimated only for those regions where more than 20 ticks were tested. The infection rates of ticks in regions where less than 20 ticks were tested were considered as hypothetical and were not included in a further analysis. The lowest virus prevalence was in the Republics of Tuva, Karelia and Tatarstan and in the Kaliningrad Region (location 1) (Table 2). The infection rates of ticks in those regions were 0.6%, 0.8%, 1.4% and 1.8%, respectively. The virus prevalence in other regions ranged from 3.8% (the Republic of Altai) to 7.8% (location 3 in the Kaliningrad Region) (Table 2).

Four randomly selected ALSV-positive samples: Miass502, Miass506, Miass15-T22516 and Miass15-T22517 were isolated in the IRE/CTVM19 cell line, in addition to the three previously reported strains: Miass519, Miaas527 and Galozero-14-T20426 [7]. These seven strains are marked with the letter “s” in Table 1. None of the strains caused any cytopathic effect in IRE/CTVM19 cells. In addition, we tested the HAE/CTVM8 cell line for the ability to support the replication of strain Miass527. This strain successfully replicated in HAE/CTVM8 cells over 14 months without cytopathic effect.

**Figure 3:**
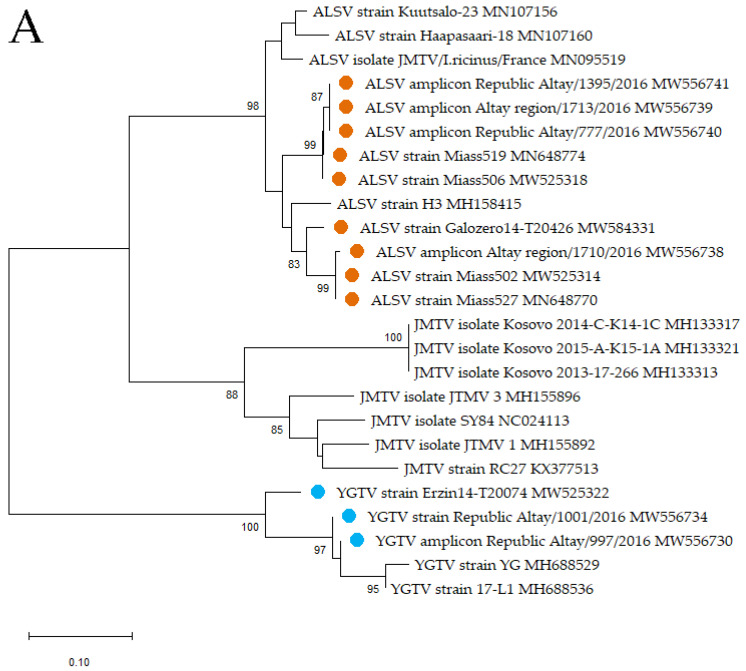

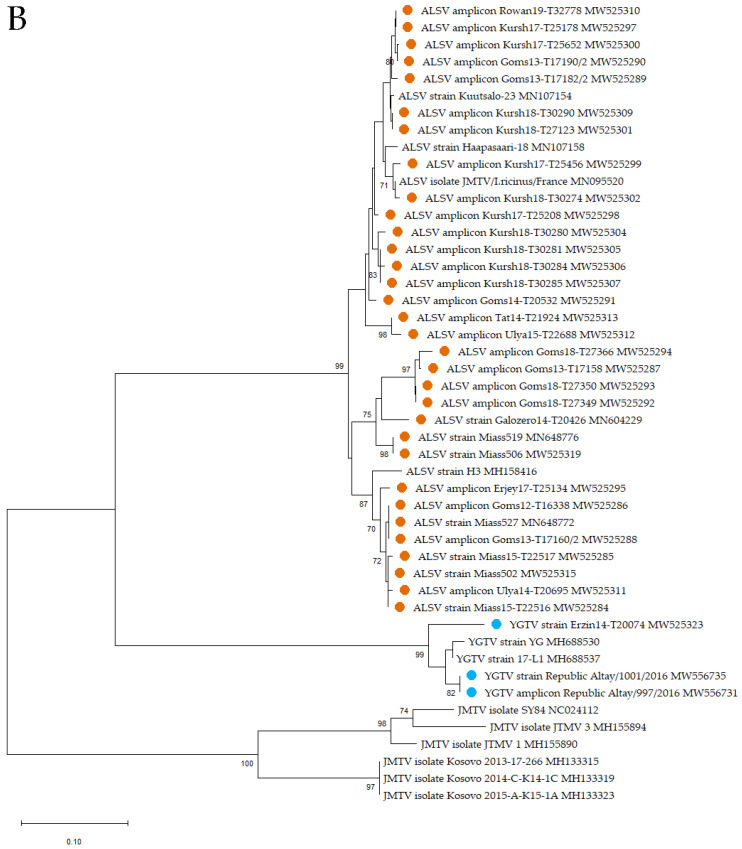
Phylogenetic trees of the Alongshan and Yanggou tick viruses. Phylogenetic trees were constructed using the neighbour-joining method in MEGA X (1000 bootstrap replications). Bootstrap values (>70%) are shown at the branches. GenBank accession numbers are listed for each strain/isolate/amplicon. Descriptions of the sequences published in GenBank were reproduced without changes. Orange circles—amplicons/strains of the Alongshan virus (ALSV) described in this study. Blue circles—amplicons/strains of the Yanggou tick virus (YGTV) described in this study. (**A**) Phylogenetic tree of a 188-bp fragment of segment 1 of ALSV, YGTV and the Jingmen tick virus (JMTV). (**B**) Phylogenetic tree of a 233-bp fragment of segment 2 of ALSV, YGTV and JMTV.

For the phylogenetic analysis, we used all published homologous RNA sequences of ALSV, including the amplicons/strains described in this article. The phylogenetic analysis was performed on a 188-bp fragment of segment 1 (Figure 3A) and on a 233-bp fragment of segment 2 (Figure 3B). All of our strains clustered for both segments with other ALSV. The branching of the phylogenetic trees obtained from the analysis of the genome fragments of segments 1 and 2 is almost identical. However, in the analysis of segment 2, the strain Galozero14-T20426 is clustered with the strains Miass519 and Miass506, while in the analysis of segment 1, it is clustered with strain H3. This may be due to reassortment or to the fact that the analysis was carried out on short fragments.

**Figure 4:**
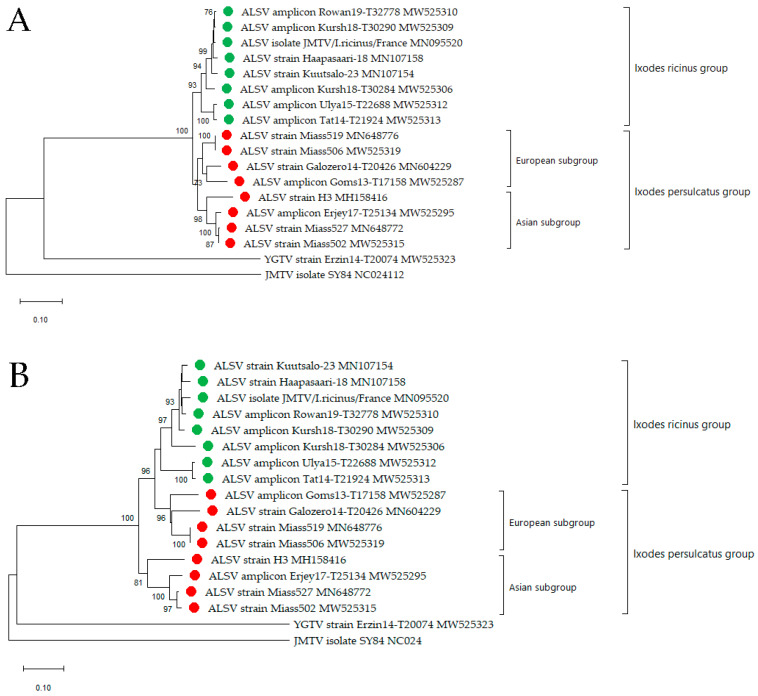

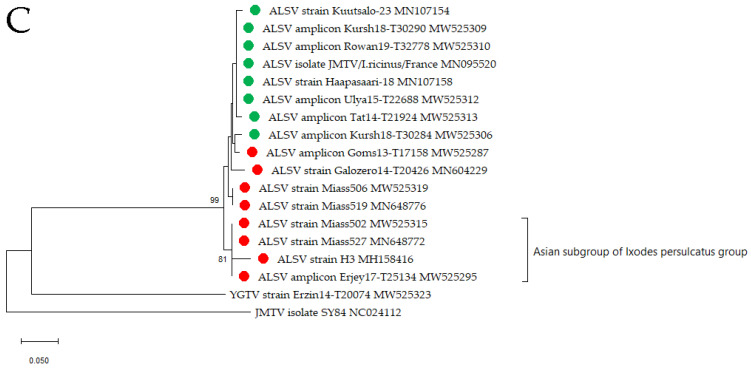
Phylogenetic analysis of the Alongshan virus (ALSV) proteins was performed using the neighbour-joining method in MEGA X. Descriptions of the sequences published in GenBank were reproduced without changes. Red circle—amplicons/strains from the *Ixodes persulcatus* group, and green circle—amplicons/strains from the *Ixodes ricinus* group (**A**). Complete amino acid sequence of the VP1a protein. (**B**) Complete amino acid sequence of the VP1b protein. (**C**) Complete amino acid sequence of the novel upstream open reading frame (nuORF) protein. YGTV = Yanggou tick virus and JMTV = Jingmen tick virus.

Segment 2 encodes VP1a, VP1b and the previously described nuORF [7] proteins. Since VP1a and VP1b are believed to be surface glycoproteins [5,6], we assumed that these proteins were responsible for the association with the vector species. For two strains, Miass502 and Miass506, high-throughput sequencing of the virus purified from the IRE/CTVM19 cell supernate was carried out in order to obtain full genomes, while for eight positive tick samples, partial sequences of segment 2 were obtained via Sanger sequencing using primers targeting segment 2 (Table S3). We performed a phylogenetic analysis of the amino acid sequences of the VP1a, VP1b and nuORF proteins of segment 2 (Figure 4A–C).

The VP1a phylogenetic analysis divided amplicons/strains by tick species into *I. ricinus* and *I. persulcatus* groups, and then, the *I. persulcatus* group was further divided into European and Asian subgroups (Figure 4A). Strains Galozero14-T20426 and Coms13-T17158 isolated from the European part of Russia and two strains Miass519 and Miass506 from the Chelyabinsk Region created the European *I. persulcatus* subgroup, separate from strains Erjey17-T25134 (the Republic of Tuva), H3 (China) and two strains Miass527 and Miass502 from the Chelyabinsk Region, which formed the Asian *I. persulcatus* subgroup.

According to the phylogenetic analysis of the VP1b protein, the Asian *I. persulcatus* subgroup was first separated from a European group, which was further divided according to tick species, as it included the European *I. persulcatus* subgroup and the *I. ricinus* group (Figure 4B).

The phylogenetic analysis of the nuORF sequences showed the presence of the Asian *I. persulcatus* subgroup, but there was no support for any other groups (Figure 4C). This may be due to the fact that the nuORF sequence is short and highly conserved.

We studied the differences in the amino acid sequences of the proteins nuORF, VP1a and VP1b in the *I. ricinus* and *I. persulcatus* groups. In the VP1a protein, we found seven substitutions that were specific for the *I. ricinus* and *I. persulcatus* groups. These substitutions were found in amino acid positions 72, 115, 135, 138, 153, 216 and 472 of the protein (Table 3). In VP1b, we found only one such substitution in amino acid position 276 (Table 3). There were no specific amino acid differences between the *I. ricinus* and *I. persulcatus* groups in the nuORF protein.

We then compared the amino acid sequences of the nuORF, VP1a and VP1b proteins of the European and Asian *I. persulcatus* subgroups. We found three specific amino acid substitutions in positions 4, 15 and 132 in the nuORF; six specific amino acid substitutions in positions 8, 210, 321, 460, 476 and 479 in the VP1a protein and four specific amino acid substitutions in positions 118, 172, 187 and 195 in the VP1b protein (Table 3).

### 3.3. Yanggou Tick Virus Detection, Isolation and Phylogenetic Relationships

We detected YGTV in three samples of ticks collected in two neighbouring republics: the Republic of Tuva and the Republic of Altai in the Asian part of Russia (Figure 2 and Table 1). YGTV was detected in a pool of *D. nuttalli* ticks removed from animals (cattle) and in two individual *D. marginatus* ticks collected from vegetation using panflavi primers.

The virus prevalence was very different between the two neighbouring regions, being 0.5% in the Republic of Tuva and 8.0% in the Republic of Altai (Table 2).

Two YGTV strains, Erzin14-T20074 and Republic Altay/1001/2016, were successfully isolated in the IRE/CTVM19 cell culture and persisted for over 12 months without cytopathic effect. We also used the HAE/CTVM8 cell culture for propagation of the strain Erzin14-T20074, which successfully replicated in the HAE/CTVM8 cells over eight months without cytopathic effect. For two strains and one amplicon of YGTV, high-throughput sequencing was carried out in order to obtain full genomes.

For the phylogenetic analysis, we used RNA sequences of all published strains of YGTV, including the strains/amplicon described in this article. The phylogenetic analysis was performed on a 188-bp fragment of segment 1 and on a 233-bp fragment of segment 2. All of our strains clustered with other YGTV. At the same time, strain Erzin14-T20074 from *D. nuttalli* collected in the Republic of Tuva formed a separate branch within the YGTV group, and strain Republic Altay/1001/2016 and amplicon Republic Altay/997/2016 from *D. marginatus* collected in the Republic of Altai clustered with the strains from China (Figure 3A,B).

## 4. Discussion

The first isolation of JMTV was carried out in China from *Rhipicephalus microplus* ticks [3]. A large number of the JMV group representatives have been detected in invertebrate [2,5,11,14] and vertebrate [10,11,12,18,20,22] hosts in many countries and continents [2,5,11,15,16,17,19]. JMTVs, YGTVs and ALSVs are included in the JMV group, along with other segmented flavi-like viruses.

Representatives of the JMV group were detected China in *Culex tritaeniorhynchus* and *Anopheles yatsushiroensis* mosquitoes [20]. However, we did not detect any flavi-like viruses in the mosquitoes collected in the western part of Russia.

Sequences of YGTV from 21 samples or strains obtained from ticks collected in the Xinjiang Uygur Autonomous Region of China were first deposited in GenBank in July 2018 (accession numbers MH688529.1-MH688539.1 from three strains derived from *D. nuttalli* ticks and MH688679.1-MH688696.1 from 18 strains derived from unspecified tick species). The phylogenetic analysis of the partial sequences of segments S1 and S3 revealed that YGTV is closely related to ALSV, and both viruses cluster separately from other JMV group viruses [22].

We detected three isolates of YGTV in two neighbouring regions of the Central Asian part of Russia. Strain Republic Altay/1001/2016 and amplicon Republic Altay/997/2016 were detected in questing *D. marginatus*. Since *D. marginatus* ticks can be found not only in Russia and China but also in Europe and North Africa [36], we can expect the circulation of this virus in other countries. The strain Erzin14-T20074 was isolated from engorged *D. nuttalli* ticks removed from cattle. We cannot exclude the possibility that YGTV was obtained during tick feeding on the bovine host, as viremia of other representatives of the JMV group have been detected during mammalian infections [12,20,22]. For the first time, we demonstrated the successful replication of YGTV in the IRE/CTVM19 and HAE/CTVM8 tick cell lines.

The first detection of ALSV was in China from a patient suffering from a fever of unknown aetiology [20]. Subsequently, this virus was detected in *I. persulcatus* [7,20] and *I. ricinus* [11,13] ticks and in mosquitoes [20], sheep and cattle [22]. We detected ALSV in different regions of Russia in 44 samples of individual or pooled unfed *I. persulcatus*, *I. ricinus*, *D. reticulatus*, *D. nuttalli* and *H. concinna* ticks but not in mosquitoes. The detection of ALSV over a large area, from the Kaliningrad Region in Europe to the Republic of Tuva in Asia, indicates that this virus may be widely distributed in Russia. This is especially important in view of the fact that there is information that ALSV can cause disease in humans [20].

Seven ALSV strains, including three reported previously [7], and two YGTV strains successfully replicated in IRE/CTVM19 tick cells form persistent infections. In addition, the *I. persulcatus*-derived ALSV strain Miass527 and the *D. nuttalli*-derived YGTV strain Erzin14-T20074 form persistent infections in the HAE/CTVM8 cell line. The reproduction of ALSV and YGTV in cell lines of different tick species could indicate that various tick species can be vectors or that these viruses can rapidly adapt to different tick species. For example, the tick-borne encephalitis virus (TBEV) can successfully replicate in *Dermacentor* ticks, whose competence as natural TBEV vectors is under discussion [32,37,38], and even in *H. marginatum* [39], which is not known to be a vector of this virus. On the other hand, tick cell lines can support the replication of a wide range of tick- and insect-borne arboviruses that are not known to be transmitted by the parent tick species [40].

According to the phylogenetic analysis carried out both on genome fragments of segments 1 and 2 and on the complete amino acid sequences of the VP1a proteins (segment 2), ALSV can be divided into two clades: an *I. persulcatus* clade and an *I. ricinus* clade. However, the phylogenetic analysis of VP1b showed the presence of Asian and European clades, the latter of which was divided into the “*I.* *ricinus*” and “*I. persulcatus*” groups. Regardless of which gene or protein was used in the phylogenetic analysis, strains isolated from the areas of absolute dominance by *I. persulcatus* (with the absence of *I. ricinus*) or by *I. ricinus* (without the presence of *I. persulcatus*) formed separate groups. The *I. persulcatus* areas include the Republics of Tuva [41] and Altai [42], Altai Territory, Chelyabinsk Region [43] and China [44], whereas *I. ricinus* inhabits the Kaliningrad Region [45] and Europe, including France [46].

The division of ALSV isolates into groups is not well-defined in the sympatric zone occupied by both *I. ricinus* and *I. persulcatus* ticks. It has been assumed that the zone of sympatry of these tick species runs through the territories of the Republic of Tatarstan, Ulyanovsk Region, the Republic of Karelia [47] and Finland [48]. However, ALSV was detected in an area inhabited only by *I. ricinus* ticks in Finland [13] and only by *I. persulcatus* in the Republic of Karelia [7,47]. Two Finnish strains detected in *I. ricinus* [13] belonged to the *I. ricinus* group. A slightly different situation was observed in the Republic of Karelia, where ten ALSV amplicons were detected in *I. persulcatus* ticks. Six amplicons and one isolated strain were found to belong to the *I. persulcatus* group and three amplicons: Goms13-T17182/2, Goms13-T17190/2 and Goms14-T20532 to the *I. ricinus* group. This may be due to the fact that short sequences were used in the phylogenetic analysis. However, if these three amplicons actually belong to the *I. ricinus* group, then this could have happened as a result of mammals or birds introducing the virus into the territory in which *I. ricinus* ticks have never before been detected. Additionally, we cannot exclude the possibility of ALSV belonging to the *I. ricinus* group infecting *I. persulcatus* ticks and vice versa, as shown by the example of TBEV in the sympatric zone [49,50]. In the Ulyanovsk Region, we detected two positive samples in *I. ricinus* and *D. reticulatus* ticks. The amplicon Ulya15-T22688 from *I. ricinus* belongs to the *I. ricinus* clade, but the amplicon Ulya14-T20695 from *D. reticulatus* belongs to the *I. persulcatus* clade, as well as positive samples from the Republic of Altai and Altai Territory that were detected in *D. nuttalli* and *H. concinna*, respectively. Since the territories of the Republic of Altai and Altai Territory are not only inhabited by *I. persulcatus* ticks, perhaps other tick species can also receive ALSV during cofeeding on an infected animal and are not specific vectors. Further study of the possible cofeeding transmission of ALSV is needed, as well as the study of the vector competence of the *Dermacentor* and *Haemaphysalis* tick species. We can observe the same pattern in TBEV when the distribution of the European subtype is associated with *I. ricinus* ticks and of the Siberian and Far Eastern subtypes with *I. persulcatus* ticks [51].

Taken together, our results show that the ALSV strains can be divided into Asian and European subgroups and, also, that there is a clear correlation with *I. ricinus* and *I. persulcatus* ticks, respectively. Our findings differs from the data about JMTV, where geographic isolation, rather than host species, may be the main driver of JMTV diversity [52].

The ALSV *I. persulcatus* group is divided into two subgroups: European (the Republics of Karelia and Altai and Chelyabinsk Region) and Asian (China; the Republics of Altai, Tuva and Karelia; Chelyabinsk and Ulyanovsk Regions and Altai Territory). The territories of the Chelyabinsk Region and the Republics of Altai and Karelia are of particular interest, since, in these territories, strains belonging to both *I. persulcatus* subgroups were isolated. The strain Galozero14-T20426 from the Republic of Karelia appeared in different subgroups in the phylogenetic analysis of various segments, possibly indicating reassortment, as shown for JMTV [3]. The allocation of strains from the Republic of Altai to the European and Asian subgroups may also indicate reassortment or the spread of the European *I. persulcatus* subgroup to the Republic of Altai. We found specific amino acid signatures that allowed us to distinguish between the *I. ricinus* and *I. persulcatus* groups and European and Asian *I. persulcatus* subgroups. These observations, as well as the fact that isolates from various tick species belong either to the *I. ricinus* or *I. persulcatus* groups, provide further support for the idea that the distribution of ALSV depends on tick species and not only on the territory.

## 5. Conclusions

We showed, for the first time: (1) the circulation of YGTV in the territory of Russia, (2) the widespread distribution of ALSV in Eurasia, (3) the ability of ALSV and YGTV to form persistent infections in the *I. ricinus* (IRE/CTVM19) and *H. anatolicum* (HAE/CTVM8) tick cell lines, (4) the division of ALSV strains into the *I. ricinus* and *I. persulcatus* groups and their specific amino acid signatures and (5) the division of the ALSV *I. persulcatus* group into the European and Asian subgroups and their specific amino acid signatures.

## Figures and Tables

**Table 1:** Strains (“s”) or amplicons (“a”) of the Alongshan virus and Yanggou tick virus isolated from or detected in *Ixodes persulcatus*, *Ixodes ricinus*, *Dermacentor nuttalli*, *Dermacentor reticulatus*, *Dermacentor marginatus* and *Haemaphysalis concinna* ticks collected in different regions of Russia.

Strain (s)/Amplicon (a)	Tick Species	Year, Region (GPS)	GenBank Access. No.
**Alongshan virus**			
a. Miass501	*I. persulcatus*	2014, Chelyabinsk Region (55.02145°, 60.168283°)	MT210222
s. Miass502	*I. persulcatus*		MW525314–MW525317
s. Miass506	*I. persulcatus*		MW525318–MW525321
a. Miass508	*I. persulcatus*		MT210221
a. Miass510	*I. persulcatus*		MT210225
a. Miass515	*I. persulcatus*		MT210223
s. Miass519	*I. persulcatus*		MN648774–MN648777
a. Miass523	*I. persulcatus*		MT210224
s. Miass527	*I. persulcatus*		MN648770–MN648773
s. Miass15-T22516	*I. persulcatus*	2015, Chelyabinsk Region (55.021583°, 60.169783°)	MW525284
s. Miass15-T22516	*I. persulcatus*		MW525285
a. Goms12-T16338	*I. persulcatus*	2012, Republic of Karelia (62,0690667°, 33,96141667°)	MW525286
a. Goms13-T17158	*I. persulcatus*	2013, Republic of Karelia (62.069381°, 33.964528°)	MW525287
a. Goms13-T17160/2	*I. persulcatus*		MW525288
a. Goms13-T17182/2	*I. persulcatus*	2013, Republic of Karelia (62.068557°, 33.962730°)	MW525289
a. Goms13-T17190/2	*I. persulcatus*		MW525290
s. Galozero-14-T20426	*I. persulcatus*	2014, Republic of Karelia (62.075051°, 33.951404°)	MN604229, MW584331
a. Goms14-T20532	*I. persulcatus*	2014, Republic of Karelia (62.063838°, 33.943815°)	MW525291
a. Goms18-T27349	*I. persulcatus*	2018, Republic of Karelia (62.075230°, 33.946687°)	MW525292
a. Goms18-T27350	*I. persulcatus*		MW525293
a. Goms18-T27366	*I. persulcatus*	2018, Republic of Karelia (62.067203°, 33.933651°)	MW525294
a. Erjey17-T25134	*I. persulcatus*	2017, Republic of Tuva (51.32646°, 95.98224°)	MW525295
a. Sizim17-T25125	*I. persulcatus*	2017, Republic of Tuva (51.33110°, 95.94315°)	MW525296
a. Republic Altay/777/2016	*D. nuttalli*	2016, Republic of Altai (51.24428°, 86.06387°)	MW556740
a. Republic Altay/1395/2016	*D. nuttalli*	2016, Republic of Altai (49.8006°, 88.8875°)	MW556741
a. Altay region/1710/2016	*H. concinna*	2016, Altai Territory (52.14821°, 85.97828°)	MW556738
a. Altay region/1713/2016	*H. concinna*		MW556739
a. Kursh17-T25178	*I. ricinus*	2017, Kaliningrad Region (54.972030°, 20.508669°)	MW525297
a. Kursh17-T25208	*I. ricinus*		MW525298
a. Kursh17-T25456	*I. ricinus*	2017, Kaliningrad Region (55.18242°, 20.85957°)	MW525299
a. Kursh17-T25652	*I. ricinus*	2017, Kaliningrad Region (54.97020°, 20.50458°)	MW525300
a. Kursh18-T30280	*I. ricinus*	2018, Kaliningrad Region (55.146110°, 20.824530°)	MW525304
a. Kursh18-T30274	*I. ricinus*	2018, Kaliningrad Region (55.15465°, 20.82790°)	MW525302
a. Kursh18-T27123	*I. ricinus*	2018, Kaliningrad Region (55.183363°, 20.857993°)	MW525301
a. Kursh18-T30281	*I. ricinus*	2018, Kaliningrad Region (55.175897°, 20.846458°)	MW525305
a. Kursh18-T30284	*I. ricinus*	2018, Kaliningrad Region (55.1591837°, 20.8432753°)	MW525306
a. Kursh18-T30285	*I. ricinus*		MW525307
a. Kursh18-T30286	*I. ricinus*		MW525308
a. Kursh18-T30278	*I. ricinus*	2018, Kaliningrad Region (55.222354°, 20.891556°)	MW525303
a. Kursh18-T30290	*I. ricinus*		MW525309
a. Rowan19-T32778	*I. ricinus*	2019, Kaliningrad Region (54.827417°, 20.517167°)	MW525310
a. Ulya14-T20695	*D. reticulatus*	2014, Ulyanovsk Region (54.446204°, 48.379524°)	MW525311
a. Ulya15-T22688	*I. ricinus*	2015, Ulyanovsk Region (54.588423°, 48.416590°)	MW525312
a. Tat14-T21924	*I. ricinus*	2014, Republic of Tatarstan (55.85397°, 48.7577°)	MW525313
**Yanggou tick virus**			
s. Erzin14-T20074	*D. nuttalli*	2014, Republic of Tuva (50.13304°, 95.4722°)	MW525322–MW525325
s. Republic Altay/1001/2016	*D. marginatus*	2016, Republic of Altai (52.02653°, 85.85219°)	MW556734–MW556737
a. Republic Altay/997/2016	*D. marginatus*		MW556730–MW556733

We used the following definition of the “virus strain” (“s”): a virus that was passaged several times in or persistently infected tick cell lines, which resulted in a virus population with stable properties under given laboratory conditions. By “amplicon” (“a”), we mean the primary material containing viral RNA fragments with the identified nucleotide sequences without strain isolation.

**Table 2:** Virus prevalence in Ixodes persulcatus, Ixodes ricinus, Dermacentor nuttalli, Dermacentor reticulatus, Dermacentor marginatus and Haemaphysalis concinna ticks collected in different regions of Russia.

Region of Russia	Tick Species	Number of Analysed Ticks (Total)	Number of Analysed Pools/Individual Ticks	Number of Positive Pools/Individual Ticks	MIR ^1^ (%)
**Alongshan virus**					
Republic of Tuva	*I. persulcatus*	348	73/12	2/0	0.6
Republic of Altai	*D. nuttalli*	19	0/19	0/1	5.2 ^2^
	*D. nuttalli*	26	0/26	0/1	3.8
Altai Territory	*H. concinna*	16	0/16	0/2	12.5 ^2^
Chelyabinsk Region	*I. persulcatus*	254	62/0	11/0	4.3
Republic of Tatarstan	*I. ricinus*	74	16/0	1/0	1.4
Ulyanovsk Region	*I. ricinus*	20	0/20	0/1	5.0 ^2^
	*D. reticulatus*	9	2/0	1/0	11.1 ^2^
Republic of Karelia	*I. persulcatus*	1265	265/0	10/0	0.8
Kaliningrad Region	*I. ricinus* (1) ^3^	57	23/0	1/0	1.8
	*I. ricinus* (2) ^3^	59	14/0	3/0	5.1
	*I. ricinus* (3) ^3^	129	30/0	10/0	7.8
**Yanggou tick virus**					
Republic of Tuva	*D. nuttalli*	205	49/20	1/0	0.5
Republic of Altai	*D. marginatus*	25	0/25	0/2	8.0

The table shows only data for those collections where PCR-positive samples were detected, and all negative samples were omitted. All tested ticks are presented in Table S2. ^1^ MIR (minimal infection rate) = $\frac{\mathrm{Number}\;\mathrm{of}\;\mathrm{positive}\;\mathrm{pools}\;+\;\mathrm{number}\;\mathrm{of}\;\mathrm{positive}\;\mathrm{individual}\;\mathrm{ticks}}{\mathrm{Number}\;\mathrm{of}\;\mathrm{analysed}\;\mathrm{ticks}\;\left(\mathrm{total}\right)}$. ^2^ Hypothetical data, due to the small amount of analysed ticks. ^3^ Division into collection points (see Figure S1).

**Table 3:** Specific amino acid substitutions in the Alongshan virus VP1a, VP1b and novel upstream open reading frame (nuORF) proteins detected in the *Ixodes ricinus* group and European and Asian *Ixodes persulcatus* subgroups.

Amino Acid Position	*Ixodes ricinus* Group	*Ixodes persulcatus* Group	
		European Subgroup	Asian Subgroup
**VP1a**			
8	Ala	Ala	Thr
72	Val	Ala	Ala
115	Ala	Val	Val
135	Val	Lys	Lys
138	Pro	Ser	Ser
153	Lys	Arg	Arg
210	Gly	Gly	Ser
216	Thr	Ala	Ala
321	Val	Val	Thr
460	Thr	Met	Thr
472	Arg	His	His
476	Arg	Arg	Gln
479	Arg/His	Arg	His
**VP1b**			
118	Met	Met	Leu
172	Ile	Ile	Va
187	Lys	Lys	Arg
195	Ser	Ser	Gly
276	Val	Ile	Ile
**nuORF**			
4	Lys	Lys	Gln
15	Asp	Asp	Asn
132	Thr	Ala	Thr

Orange colour—specific amino acid positions distinguishing the *I. ricinus* group and *I. persulcatus* group. Green colour—amino acid positions distinguishing the European and Asian *I. persulcatus* subgroups.

## Data Availability

The data presented in this study are available in the article.

## Supplementary Materials

The following are available online at https://www.mdpi.com/1999-4915/13/3/458/s1: Table S1: The locations of mosquito collections in regions of Russia between 2013 and 2018, species and their abundance. Table S2: The locations of tick collections and tick species in regions of Russia between 2011 and 2019 (only tested ticks are presented). Table S3: Specific primers for the amplification of segments 1 and 2 of the Alongshan virus genome. Figure S1: Locations of Alongshan virus detection in the Kaliningrad Region.
