# Supplementary material for: Geographical and Tick-Dependent Distribution of Flavi-Like Alongshan and Yanggou Tick Viruses in Russia
Source: Viruses. 2021 Mar 11;13(3):458. doi: 10.3390/v13030458 (PMC7998622; doi:10.3390/v13030458)
Supplement: Supplementary file 1 [file viruses-13-00458-s001.pdf]

Table S1. The locations of mosquito collections in regions of Russia between 2013 and 2018, species and their abundance

Latitude	Longitude	Date (mm.year)	*Ae. cantans*	*Ae. cinereus*	*Ae. communis*	*Cx. pipens*	*An. messeae*
**Komi Republic**							
61.633333°	50.716667°	08.2018	10	3	5		
61.633334°	50.716668°	09.2018	30	5			
61.633335°	50.716669°	10.2018				10	
		**Total:**	**40**	**8**	**5**	**10**	
**Kirov region**							
57.95°	48.333333°	09.2018				10	20
58.00°	48.45°	09.2018				5	30
		**Total:**				**15**	**50**

Latitude	Longitude	Date (mm.year)	*Ae. punctor*	*Ae. communis*	*Ae. pionops*	*Ae. cantans*	*Culiseta bergrothi*	*Ae. diantaeus*
**Arkhangelsk region**								
64.938245°	36.817821°	05.2016	22	32				
64.950823°	36.819048°	05.2016	47	54	26		1	
64.948676°	36.830135°	05.2016	32	191	32			
64.940555°	36.818042°	05.2016	80	196	48			
64.945608°	36.746565°	06.2016	27	249	21			
64.938312°	36.817333°	06.2016	4	377	8			
64.952440°	36.820510°	06.2016	4	93	4			
64.957893°	36.836259°	06.2016	9	234	12	1		
64.955013°	36.849614°	06.2016	25	589	37		1	
64.961213°	36.844939°	06.2016	4	89	4	2		
64.949593°	36.827714°	06.2016	20	289	18			6
		**Total:**	**274**	**2393**	**210**	**3**	**2**	**6**

Latitude	Longitude	Date (mm.year)	*Ae. cantans*	*Ae. cinereus*	*Ae. communis*	*Cx. territans*	*Ae. punctor*	*Ae. diantaeus*	*An. claviger*	*Culiseta morsitans*
**Leningrad region**										
59.722681°	30.179718°	05-06.2014		28		49			2	20
		**Total:**		**28**		**49**			**2**	**20**
**Saint Petersburg**										
59.831347°	30.138416°	04-05.2014	298	91	108		9	1		
59.841955°	30.190558°	05-06.2014	90	82	26		4	6		
59.836207°	30.046358°	05.2014			337		45			
		**Total:**	**388**	**173**	**471**		**58**	**7**		

Latitude	Longitude	Date (mm.year)	*Ae. punctor*	*Ae. communis*	*Ae. cinereus*
**The Republic of Karelia**					
62.068362°	33.959205°	06.2013		62	
62.069959°	33.965455°	06.2013	31	97	5
		**Total:**	**31**	**161**	**5**

Table S2. The locations of tick collections and tick species in regions of Russia between 2011 and 2019 (only tested ticks are presented)

Latitude	Longitude	Date (mm.year)	*I. persulcatus* (♂+♀)/NN	*I. ricinus* (♂+♀)/NN	*D. reticulatus* (♂+♀)	*D. marginatus* (♂+♀)	*D. nuttalli* (♂+♀)	*D. silvarum* (♂+♀)	*H. concinna* (♂+♀)	Method of tick collection
**The Republic of Karelia**										
62.06906667°	33.96141667°	04-06.2012	46+61							FV
62.06558333°	33.9585°	05.2012	34+39							FV
62.07098333°	33.95605°	05.2012	6+7							FV
62.06785°	33.94811667°	05.2012	7+6							FV
62.0693°	33.93928333°	05.2012	13+7							FV
62.0656°	33.93786667°	05.2012	21+22							FV

Latitude	Longitude	Date (mm.year)	*I. persulcatus* (♂+♀)/NN	*I. ricinus* (♂+♀)/NN	*D. reticulatus* (♂+♀)	*D. marginatus* (♂+♀)	*D. nuttalli* (♂+♀)	*D. silvarum* (♂+♀)	*H. concinna* (♂+♀)	Method of tick collection
62.06866667°	33.9558°	05.2012	3+2							FV
62.06558333°	33.9489°	05.2012	4+4							FV
62.0569°	33.90941667°	05.2012	30+31							FV
62.06741667°	33.9751833°	05.2012	35+39							FV
61.839716667°	34.39935°	05.2012	10+0/1							FV
61.4894833°	34.8331°	05.2012	1+1							FV
61.48428333°	34.83981667°	05.2012	8+5/1							FV
61.4715833°	34.872°	05.2012	5+9	0+1						FV
61.45285°	35.06368333°	05.2012	2+1							FV
61.3608333°	35.4412833°	05.2012	4+6	0+2						FV
61.38268333°	35.348233°	05.2012	4+8	0+5						FV
62.09286667°	35.196533°	05.2012	4+6							FV
62.09588333°	35.20738333°	05.2012	1+1							FV
62.05408333°	35.2523833°	05.2012	17+16							FV
62.1109°	35.20146667°	05.2012	9+12							FV
62.11073333°	35.16896667°	05.2012	3+1							FV
62.10736667°	35.1718333°	05.2012	3+2	1+0						FV
62.07518333°	35.203667°	05.2012	6+6							FV
62.05591667°	35.20851667°	05.2012	1+3							FV
62.48491667°	31.226433°	06.2012	4+12							FV
62.385833°	31.12685°	06.2012	6+6							FV
62.342916667°	31.00335°	06.2012	7+12							FV
62.3462667°	30.99165°	06.2012	0+6							FV
61.6718°	31.02995°	08.2012		4+6						FV
61.36638333°	30.8858667°	06.2013	4+3							FV
61.36656667°	30.8982667°	06.2013	1+1							FV
61.3603333°	30.89645°	06.2013	1+1							FV
61.39248333°	30.94158333°	06.2013	0+1							FV
61.3942°	30.94816667°	06.2013	6+5							FV
61.3997333°	30.9516333°	06.2013	12+16							FV
61.39596667°	30.95738333°	06.2013	3+6							FV
61.3946°	30.9651°	06.2013	12+8	1+2						FV
61.3667333°	30.96146667°	06.2013	1+2							FV

Latitude	Longitude	Date (mm.year)	*I. persulcatus* (♂+♀)/NN	*I. ricinus* (♂+♀)/NN	*D. reticulatus* (♂+♀)	*D. marginatus* (♂+♀)	*D. nuttalli* (♂+♀)	*D. silvarum* (♂+♀)	*H. concinna* (♂+♀)	Method of tick collection
61.36218333°	30.9273°	06.2013	1+1	1+0						FV
61.3906667°	30.91231667°	06.2013	9+16	4+5						FV
61.36636°	30.88581°	06.2013	6+6	0+2						FV
61.36095°	30.8878333°	06.2013	1+1	1+0						FV
61.388665°	30.904965°	06.2013	3+6	5+4						FV
61.392135°	30.914050°	06.2013	2+3							FV
61.391426°	30.909165°	06.2013	4+7							FV
61.3942°	30.94816667°	06.2014	3+5	3+6						FV
61.3941°	30.94817°	06.2014	2+3	0+1						FV
61.3947°	30.9654333°	06.2014	6+2	1+0						FV
61.39065°	30.9124°	06.2014	2+6	3+3						FV
61.38396667°	30.93511667°	06.2014	1+1							FV
61.38275°	30.93801667°	06.2014	0+2	0+1						FV
61.39421°	30.94825°	06.2014	4+13	1+5						FV
61.399916667°	30.95125°	06.2014	6+14	5+1						FV
61.39477°	30.96577°	06.2014	15+12	4+2						FV
61.3968°	30.99126667°	06.2014	11+4	4+5						FV
62.069306°	33.964447°	06.2014	14+16							FV
62.063057°	33.961113°	06.2014	5+13							FV
62.065657°	33.957460°	06.2014	24+21							FV
62.075051°	33.951404°	06.2014	21+12							FV
62.065236°	33.954858°	06.2014	19+19							FV
62.063259°	33.959555°	06.2014	5+0							FV
62.076495°	33.950999°	06.2014	23+26							FV
62.061621°	33.956321°	06.2014	6+7							FV
62.077452°	33.950400°	06.2014	18+23							FV
62.069905°	33.965143°	06.2014	62+69							FV
62.075230°	33.946687°	06.2014	17+10							FV
62.067203°	33.933651°	06.2014	28+10							FV
62.072465°	33.957672°	06.2014	10+5							FV
62.070669°	33.956407°	06.2014	3+3							FV
62.072395°	33.956022°	06.2014	0+6							FV
62.058623°	33.942770°	06.2014	0+11							FV

Latitude	Longitude	Date (mm.year)	*I. persulcatus* (♂+♀)/NN	*I. ricinus* (♂+♀)/NN	*D. reticulatus* (♂+♀)	*D. marginatus* (♂+♀)	*D. nuttalli* (♂+♀)	*D. silvarum* (♂+♀)	*H. concinna* (♂+♀)	Method of tick collection
61.371517°	31.699517°	06.2015	8+14							FV
61.37065°	31.71575°	06.2015	5+0							FV
61.31915°	31.861683°	06.2015	18+4							FV
61.366833°	31.603567°	06.2015	2+0							FV
61.363017°	31.634333°	06.2015	5+22							FV
62.069905°	33.965143°	06.2018	68+106							FV
62.069644°	33.956797°	06.2018	32+21							FV
62.067203°	33.933651°	06.2018	32+14							FV
62.060627°	33.938344°	06.2018	0+16							FV
62.072465°	33.957672°	06.2018	15+14							FV
		**Total:**	**877+995/2**	**38+51**						FV
**Kaliningrad region**										
54.97020°	20.50458°	08.2017		7+18						FV
54.972030°	20.508669°	08.2017		0+12						FV
54.97070°	20.51055°	08.2017		4+4						FV
54.93384°	20.50463°	08.2017		6+0						FV
55.089330°	20.733026°	08.2017		0+5						FV
55.14493°	20.81964°	08.2017		4+0						FV
55.179051°	20.863502°	08.2017		0+4						FV
55.18242°	20.85957°	08.2017		5+5						FV
55.17862°	20.84944°	08.2017		11+10						FV
55.222354°	20.891556°	05.2018		4+15						FV
55.175897°	20.846458°	05.2018		0+8						FV
54.960614°	20.503409°	05.2018		14+0						FV
55.1591837°	20.8432753°	05.2018		0+21						FV
55.15465°	20.82790°	05.2018		3+9						FV
55.183363°	20.857993°	05.2018		0+2						FV
55.1525197°	20.8441098°	05.2018		8+5						FV
55.23387°	20.92020°	05.2018		0+4						FV
54.8345°	20.507472°	04.2019		0+1						FV
54.697222°	20.1935°	04.2019		4+0						FV
54.830111°	20.507528°	04.2019		0+2						FV

Latitude	Longitude	Date (mm.year)	*I. persulcatus* (♂+♀)/NN	*I. ricinus* (♂+♀)/NN	*D. reticulatus* (♂+♀)	*D. marginatus* (♂+♀)	*D. nuttalli* (♂+♀)	*D. silvarum* (♂+♀)	*H. concinna* (♂+♀)	Method of tick collection
54.827417°	20.517167°	04.2019		2+4						FV
54.699528°	20.200278°	04.2019		2+1						FV
54.700111°	20.198167°	04.2019		1+3						FV
54.830028°	20.510083°	05.2019		7+2						FV
54.701556°	20.19975°	05.2019		2+0						FV
54.836722°	20.503028°	05.2019		0+10						FV
54.697889°	20.191778°	06.2019		3+0						FV
54.827167°	20.512583°	06.2019		2+0						FV
54.837944°	20.509722°	06.2019		5+0						FV
54.698222°	20.188139°	06.2019		4+11						FV
54.824889°	20.517806°	06.2019		0+10						FV
54.835389°	20.506361°	06.2019		2+3						FV
54.824722°	20.513417°	07.2019		7+4						FV
54.698778°	20.192417°	07.2019		1+0						FV
54.699194°	20.196056°	07.2019		2+3						FV
54.698528°	20.191444°	09.2019		4+0						FV
		**Total:**		**114+176**						
**The Republic of Tatarstan**										
55.389799°	50.752858°	05.2012	26+34		1+0					FV
55.517200°	51.882729°	05.2012	31+24	0+28	3+6					FV
55.369929°	50.578420°	05.2012	0+1							FV
55.922655°	49.178930°	05.2012		35+20	2+6					FV
55.671218°	49.119788°	05.2012		0+2						FV
55.237401°	52.009485°	08.2012			4+10					FV
55.926713°	49.195080°	09.2012		31+28	22+17					FV
55.792462°	52.433137°	09.2012			10+20					FV
55.836229°	48.958919°	09.2012		6+15	14+16					FV
56.414656°	52.968641°	09.2012			0+6					FV
55.85397°	48.7577°	08.2014		3+19						FV
55.84747°	48.76821°	09.2015		8+23						FV
55.963800°	49.094880°	09.2015		10+7						FV
		**Total:**	**57+59**	**93+142**	**56+81**					**FV**

Latitude	Longitude	Date (mm.year)	*I. persulcatus* (♂+♀)/NN	*I. ricinus* (♂+♀)/NN	*D. reticulatus* (♂+♀)	*D. marginatus* (♂+♀)	*D. nuttalli* (♂+♀)	*D. silvarum* (♂+♀)	*H. concinna* (♂+♀)	Method of tick collection
**Voronezh region**										
51.713752°	39.363377°	09.2017		1+0						FV
51.630165°	39.671455°	09.2017		10+3						FV
51.630244°	39.671328°	09.2017		0+3						FV
51.630537°	39.671662°	05.2018		0+22						FV
51.667655°	39.751543°	05.2018		0+6						FV
51.638259°	39.685970°	05.2018		0+23						FV
51.6763766°	39.2741188°	05.2018		5+11						FV
51.785517°	39.406302°	05.2018		0+5						FV
51.656365°	39.036735°	09.2018		1+0						FV
51.630302°	39.671266°	09.2018		1+0						FV
51.6162239°	38.9850229°	09.2018		0+8						FV
		**Total:**		**18+81**						**FV**
**Chelyabinsk region**										
54.083520°	59.546226°	05.2014	2+1		9+8	2+10				FV
54.077678°	59.557866°	05.2014			0+3	0+2				FV
54.055812°	59.605618°	05.2014	1+7		0+2	5+12				FV
54.050145°	59.538399°	05.2014				9+15				FV
54.133333°	59.533333°	05.2014	1+0			1+7				FV
54.116667°	59.533333°	05.2014	1+0							FV
54.124452°	59.546685°	05.2014	29+30		0+5	6+3				FV
53.874638°	59.229960°	05.2014			5+4	30+30				RA
53.855183°	59.240433°	05.2014			22+36	33+55				FV
53.880754°	59.241434°	05.2014				24+48				RA
53.857781°	59.242444°	05.2014			0+3	2+2				FV
53.881783°	59.1628°	05.2014			50+106	18+33				FV
53.881103°	59.163164°	05.2014			21+45	1+2				FV
54.131565°	59.541217°	05.2014	15+8			0+1				FV
53.674117°	59.731317°	05.2014			3+1	21+25				FV
53.599617°	59.82275°	05.2014				0+2				FV
53.524360°	59.799737°	05.2014				9+27				RA
53.5159°	59.775067°	05.2014			2+0	38+63				FV

Latitude	Longitude	Date (mm.year)	*I. persulcatus* (♂+♀)/NN	*I. ricinus* (♂+♀)/NN	*D. reticulatus* (♂+♀)	*D. marginatus* (♂+♀)	*D. nuttalli* (♂+♀)	*D. silvarum* (♂+♀)	*H. concinna* (♂+♀)	Method of tick collection
53.382567°	59.9295°	05.2014	0+1		3+8	17+22				FV
53.3565°	60.377167°	05.2014				2+3				RA
52.885633°	60.051833°	05.2014	1+1		41+84	34+78				FV
52.940933°	59.935533°	05.2014			4+3					FV
52.053183°	59.957667°	05.2014			3+3	39+91				FV
53.124567°	59.896933°	05.2014			2+3	1+2				FV
53.14555°	59.935283°	05.2014	4+0		5+4	0+2				FV
53.315983°	60.1329°	05.2014			1+0					FV
53.233183°	60.535117°	05.2014	0+1		2+5	0+1				FV
52.82475°	60.5678°	05.2014				0+2				FV
52.428583°	60.306967°	05.2014				0+1				FV
52.49865°	60.14015°	05.2014				5+1				RA
52.497667°	60.00035°	05.2014			11+10	10+20				FV
52.47595°	59.871383°	05.2014			1+13	1+5				FV
52.459233°	60.249483°	05.2014			1+2	22+31				FV
52.541683°	60.394067°	05.2014			0+1					FV
54.446533°	60.79435°	05.2014				3+3				FV
54.629017°	60.650567°	05.2014	1+1			0+3				FV
55.128629°	60.072396°	05.2014	19+24							FV
55.02145°	60.168283°	05.2014	12+11/2							FV
55.021061°	60.171371°	05.2014	63+67/11							FV
54.410216°	62.698490°	05.2014			21+38	11+16				FV
55.021583°	60.169783°	05.2015	30+42/5							FV
55.01885°	60.165117°	05.2015	6+5							FV
		**Total:**	**185+199/18**		**202+383**	**274+509**				**FV**
					**5+4**	**70+109**				**RA**
**Arkhangelsk region**										
64.952197°	36.833553°	05.2016	0+1							RA
64.948619°	36.830435°	05.2016	1+0							FV
64.94961°	36.82729°	05.2016	1+1/1							FV
64.94122°	36.81999°	05.2016	1+0							FV
64.93824°	36.81782°	06.2016	0+1							FV

Latitude	Longitude	Date (mm.year)	*I. persulcatus* (♂+♀)/NN	*I. ricinus* (♂+♀)/NN	*D. reticulatus* (♂+♀)	*D. marginatus* (♂+♀)	*D. nuttalli* (♂+♀)	*D. silvarum* (♂+♀)	*H. concinna* (♂+♀)	Method of tick collection
		**Total:**	**3+2/1**							**FV**
			**0+1**							**RA**
**The Republic of Tuva**										
51.721639°	95.427194°	06.2014	1+1							RA
52.397278°	96.090889°	06.2014					0+2	0+1		FV
52.402028°	96.462194°	06.2014					2+1	0+3		FV
52.405533°	96.466033°	06.2014						2+0		FV
52.423317°	96.39225°	06.2014						0+1		FV
52.4377°	96.351233°	06.2014						0+1		FV
52.51747°	96.10324°	06.2014	0+1				0+2	0+5		RA
52.445583°	96.236806°	06.2014					0+1	0+6		RA
51.9156°	94.1475°	06.2014	1+1							FV
51.972783°	94.094883°	06.2014	3+4							FV
50.133049°	95.472203°	06.2014					2+2	2+0		RA
51.141717°	94.489633°	06.2014	0+1							FV
51.13885°	94.48575°	06.2014	8+5							FV
51.134567°	94.50455°	06.2014	7+6							FV
51.129983°	94.504633°	06.2014	9+4							FV
51.2995°	92.8313°	06.2014	56+57							FV
51.111767°	93.655467°	06.2014	1+0							FV
51.250983°	93.862867°	06.2014	1+0							FV
51.713886°	93.711729°	04.2017					1+1			FV
50.365861°	90.482528°	04.2017					65+128			FV
51.317058°	94.494942°	04.2017					74+128			FV
51.359437°	94.439276°	04.2017					12+22			FV
51.321559°	94.553314°	04.2017					33+61			FV
51.358841°	94.785403°	04.2017					6+13			FV
51.268500°	95.579506°	05.2017	3+1							FV
51.261984°	95.604054°	05.2017	5+0							FV
51.258095°	95.622221°	05.2017	0+10							FV
50.689066°	95.422158°	05.2017	0+2							FV
50.713323°	95.518628°	05.2017	3+4							FV

Latitude	Longitude	Date (mm.year)	*I. persulcatus* (♂+♀)/NN	*I. ricinus* (♂+♀)/NN	*D. reticulatus* (♂+♀)	*D. marginatus* (♂+♀)	*D. nuttalli* (♂+♀)	*D. silvarum* (♂+♀)	*H. concinna* (♂+♀)	Method of tick collection
51.32646°	95.98224°	05.2017	120+81/13							FV
51.32981°	95.93326°	05.2017	1+1							FV
51.33110°	95.94315°	05.2017	11+10							FV
51.35123°	95.84220°	05.2017	34+20							FV
51.38096°	95.68465°	05.2017	3+0				0+1	0+1		FV
51.62835°	94.42929°	05.2017					25+8			RA
52.07425°	94.038944°	05.2017	2+2				0+3			FV
52.202361°	93.832778°	05.2017	0+1							FV
52.22275°	93.851917°	05.2017	1+0							FV
52.239556°	93.911694°	05.2017	6+7							FV
52.297694°	93.931722°	05.2017					0+1			FV
52.295778°	93.921444°	05.2017	1+2							FV
50.73848°	95.30313°	05.2017	2+1							FV
50.106471°	95.139732°	05.2017					0+2			FV
50.10238°	95.14756°	05.2017					51+77			FV
50.07060°	95.87790°	05.2017					3+6			FV
50.05430°	95.81582°	05.2017					11+10			RA
50.048918°	95.797568°	05.2017					3+5			FV
51.30129°	94.84033°	05.2017					6+14			FV
51.86633°	94.26665°	05.2017					6+13			FV
51.91506°	94.15789°	05.2017	0+1							FV
51.95900°	94.11727°	05.2017	2+2							FV
51.97277°	94.09566°	05.2017	1+1							FV
**Total:**			**281+224/13**				**264+480**	**4+7**		**FV**
			**1+2**				**36+21**	**0+11**		**RA**
**Ulyanovsk region**										
54.335019°	48.278900°	04.2014			0+10					FV
54.258148°	49.736087°	04.2014		0+1						FV
54.180614°	49.382883°	04.2014			2+0					FV
54.266489°	49.711704°	04.2014			2+1					FV
54.266708°	49.712709°	04.2014	3+1							FV
54.295214°	47.252101°	04.2014			0+1	1+0				FV

Latitude	Longitude	Date (mm.year)	*I. persulcatus* (♂+♀)/NN	*I. ricinus* (♂+♀)/NN	*D. reticulatus* (♂+♀)	*D. marginatus* (♂+♀)	*D. nuttalli* (♂+♀)	*D. silvarum* (♂+♀)	*H. concinna* (♂+♀)	Method of tick collection
53.025278°	46.981400°	04.2014			4+6					FV
53.786425°	46.409684°	04.2014			3+2					FV
53.811239°	46.295426°	04.2014			3+2					FV
53.721856°	48.391140°	04.2014			1+0	0+1				FV
54.322859°	47.636575°	05.2014		0+1	0+1					FV
53.997682°	48.722627°	05.2014			6+0					FV
54.693403°	48.180755°	05.2014				0+2				FV
53.491635°	47.691051°	05.2014				3+2				FV
54.588423°	48.416590°	05.2014		1+0						FV
54.578392°	46.776101°	05.2014		0+1						FV
54.430074°	46.769602°	05.2014			0+2					FV
54.352651°	48.848778°	05.2014			1+1					FV
54.628633°	48.925963°	05.2014			1+4					FV
54.628984°	48.926714°	05.2014			4+11					FV
54.494871°	46.716315°	05.2014			0+1	2+4				FV
54.206611°	48.066107°	04.2015		0+1						FV
53.519610°	46.922686°	04.2015		0+1						FV
54.58599°	48.41638°	05.2015		9+3						FV
54.207716°	48.065584°	05.2015		0+1						FV
54.599476°	48.427208°	05.2015		1+1						FV
54.326011°	47.639686°	05.2015		8+0						FV
54.572987°	48.324509°	05.2015		3+3/2						FV
54.21031°	48.06427°	07.2015		0+1						FV
54.205522°	48.067472°	09.2015		1+2						FV
54.32334°	47.64586°	09.2015		5+0						FV
		**Total:**	**3+1**	**33+17/2**	**42+54**	**9+16**				**FV**
**Belgorod region**										
50.716118°	35.738839°	07.2013		3+3/7						FV
50.717410°	35.779407°	07.2013		13+11/60						FV
		**Total:**		**11+14/67**						**FV**
**The Republic of Altai**										

Latitude	Longitude	Date (mm.year)	*I. persulcatus* (♂+♀)/NN	*I. ricinus* (♂+♀)/NN	*D. reticulatus* (♂+♀)	*D. marginatus* (♂+♀)	*D. nuttalli* (♂+♀)	*D. silvarum* (♂+♀)	*H. concinna* (♂+♀)	Method of tick collection
51.24428°	86.06387°	04.2016					0+19			FV
51.10621°	86.15026°	04.2016						8+10		FV
51.88476°	86.0574°	04.2016						0+50	8+0	FV
52.02653°	85.85219°	04.2016			25+27	25+0				FV
51.85657°	86.12889°	04.2016						0+50		FV
49.80060°	88.88750°	04.2016					26+0			FV
		**Total:**			**25+27**	**25+0**	**26+19**	**8+110**	**8**	**FV**
**Altai region**										
52.14821°	85.97828°	05.2016			1+0			1+0	16+0	FV
52.31287°	85.91566°	05.2016							1+0	FV
		**Total:**			**1+0**			**1+0**	**17+0**	**FV**

Latitude	Longitude	Date (mm.year)	*Ha. punctata* (♂+♀)	*D. reticulatus* (♂+♀)	*D. marginatus* (♂+♀)	*H. scupense* (♂+♀)	*H. marginatum* (♂+♀)	*Rh. rossicus* (♂+♀)	*Rh. sanguineus* (♂+♀)	*I. ricinus* (♂+♀)	Method of tick collection
**Stavropol region**											
45.488461°	41.184730°	04.2011		4+2							RA
43.873866°	42.709235°	04.2011			10+42						RA
43.872368°	42.710562°	04.2011	2+8								RA
44.450204°	42.097278°	04.2011			0+4						RA
44.419586°	42.109615°	04.2011	0+5								RA
43.903336°	42.712826°	04.2011			0+5						RA
44.105218°	43.292382°	05.2011			2+7						RA
44.107735°	43.264091°	05.2011								0+19	RA
45.024704°	41.982376°	05.2011		1+5							RA
43.905786°	42.658584°	05.2011			11+22						RA
43.895102°	42.726626°	05.2011								56+85	FV
44.028835°	43.935219°	06.2011		1+0							RA
44.223752°	43.194183°	06.2011						7+15			RA
45.454190°	41.035556°	04.2011		1+1							RA

Latitude	Longitude	Date (mm.year)	*Ha. punctata* (♂+♀)	*D. reticulatus* (♂+♀)	*D. marginatus* (♂+♀)	*H. scupense* (♂+♀)	*H. marginatum* (♂+♀)	*Rh. rossicus* (♂+♀)	*Rh. sanguineus* (♂+♀)	*I. ricinus* (♂+♀)	Method of tick collection
45.483025°	41.216506°	04.2011		1+1							RA
45.821667°	43.044855°	04.2011					12+11				RA
45.639643°	42.544519°	04.2011			0+1						RA
45.818366°	43.045379°	04.2011					3+1				RA
44.747925°	43.453882°	04.2011			0+1						RA
45.381930°	44.202476°	04.2011						39+23			RA
44.841848°	43.286457°	05.2011					37+36				RA
45.826965°	42.519903°	05.2011					0+6				RA
45.098673°	43.464673°	05.2011					0+3				RA
44.442003°	43.134226°	05.2011	3+24								RA
44.443321°	43.133153°	05.2011			1+2						RA
44.235451°	43.121724°	05.2011		4+12							RA
45.334718°	41.804750°	05.2011					1+4				RA
43.868945°	42.751116°	05.2011	1+21								RA
45.818750°	43.052160°	05.2011					25+24				RA
43.866137°	42.747644°	05.2011		6+0							RA
43.866013°	42.747923°	05.2011			13+10						RA
44.314370°	44.192254°	06.2011							7+0		RA
43.874445°	42.710156°	06.2011			0+9						RA
45.360997°	44.231937°	07.2011					50+18				RA
45.356761°	44.232749°	07.2011					7+16				RA
44.445011°	42.358616°	07.2011					11+7				RA
45.034076°	44.386880°	07.2011			0+1						RA
44.367597°	43.706017°	04.2011	1+5								RA
45.441039°	41.047463°	04.2011				0+1					RA
45.439555°	41.047220°	04.2011		0+3							RA
45.422797°	41.373202°	04.2011			11+18						RA
45.544169°	41.320893°	04.2011			0+5						RA
45.497054°	41.212180°	04.2011			3+6						RA
45.655817°	41.197900°	04.2011		1+3							RA
45.570723°	41.021938°	04.2011			8+4						RA
45.836161°	41.516780°	04.2011			0+4						RA
45.394970°	41.343263°	04.2011			15+13						RA

Latitude	Longitude	Date (mm.year)	*Ha. punctata* (♂+♀)	*D. reticulatus* (♂+♀)	*D. marginatus* (♂+♀)	*H. scupense* (♂+♀)	*H. marginatum* (♂+♀)	*Rh. rossicus* (♂+♀)	*Rh. sanguineus* (♂+♀)	*I. ricinus* (♂+♀)	Method of tick collection
45.395147°	41.344887°	04.2011			70+68						FV
44.600068°	41.925142°	04.2011			7+21						RA
44.601316°	41.960214°	04.2011		15+30							RA
44.637751°	41.917067°	04.2011			3+17						RA
45.348883°	41.856729°	04.2011	0+3								RA
45.346224°	41.857286°	04.2011		2+2							RA
45.344891°	41.858378°	04.2011	2+4								RA
44.420595°	42.394630°	04.2011			5+7						RA
**Total:**					**70+68**					**56+85**	**FV**
			**9+70**	**36+59**	**89+199**	**0+1**	**135+119**	**46+38**	**7+0**	**0+19**	**RA**

RA – remove from animal

FV – flagging vegetation

♂ - adult male, ♀ - adult female, NN - nymphs

Table S3. Specific primers for amplification of segments 1 and 2 of the Alongshan virus genome.

Name of primers	Nucleotide sequence	Primer direction	Genome locus	Amplicon size, bp	Temperature, ^{0}C
Alongshan_seg1_5	AAGTGCAACAGTTGAGGGTC	forward	segment 1	800	52
Miass_NS5_1R	CCTTTTGGGTGTAGGGGACC	reverse			
Alongshan-OUT -F	AAAGRGGGAAGGARGAGTGGA	forward	segment 1	425	
Alongshan-OUT -R	TCTGTCYTTCCTCCTCTCTGCCA	reverse			
Miass_VP1a_F3	GCTTGTAAAGCTAGCGACTGGA	forward	segment 2	1315	53
Miass_gly_3R	TCACCGTCACAGTGGAATGG	reverse			
Miass_gly_2F	GATTACCCACTGGGCAGGAC	forward	segment 2	682	54
Miass_gly_1R	ACCAGGTTGGTCAAGGCAAT	reverse			
Miass_gl_1F	CCACATCACGGGAGGTATCG	forward	segment 2	1242	51
Alongshan_seg2_3	CGGGCTAACTCTCTCACTTG	reverse			

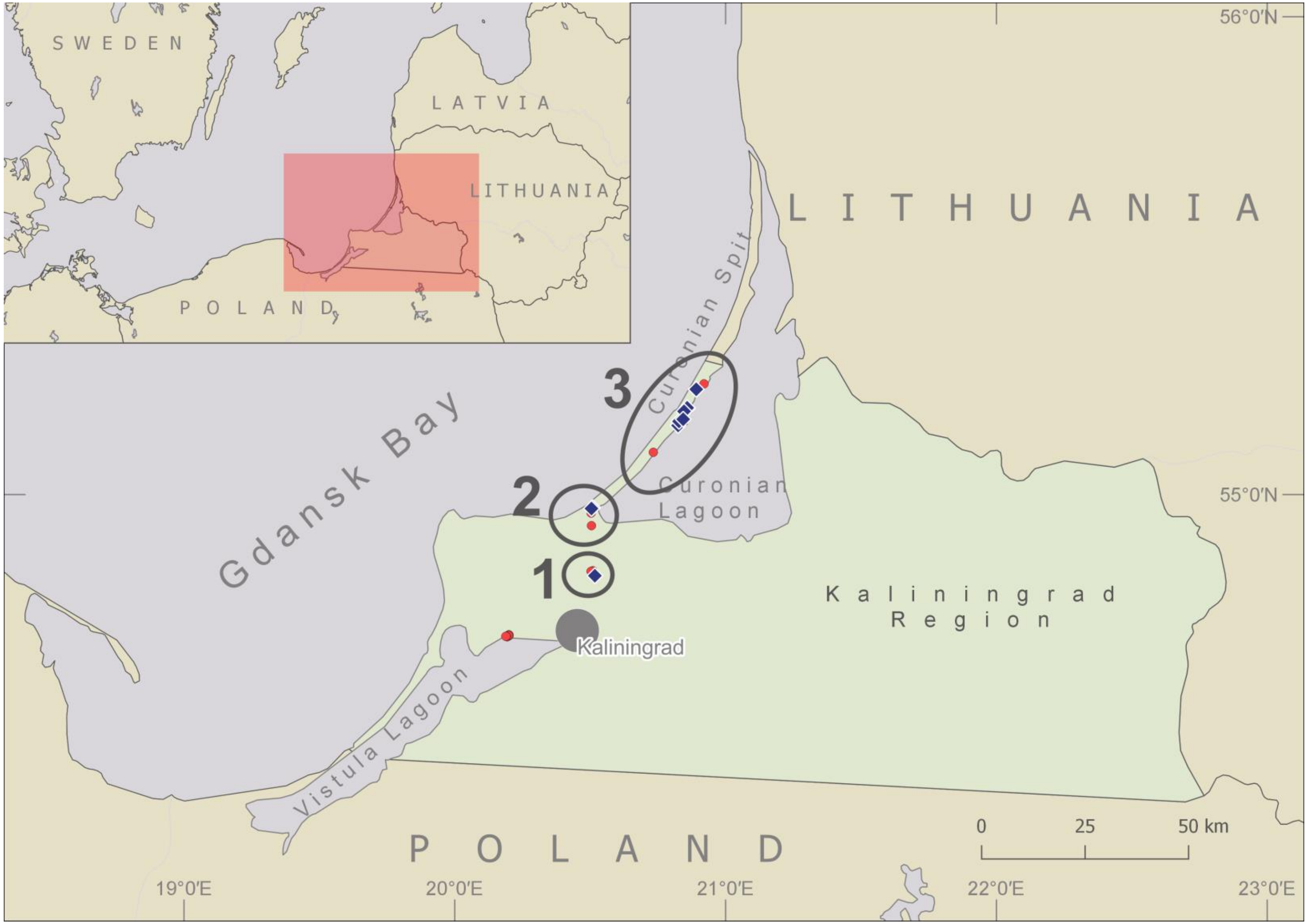

Figure S1. Locations of Alongshan virus detection in Kaliningrad region.

Red dots – locations of *Ixodes ricinus* collection; Blue diamonds - locations of Alongshan virus detection.

Black circles - three collection points that correspond to the numbers in Table 2, line Kaliningrad region.
